# Prefrontal engagement predicts the effect of museum visit on psychological well-being: an fNIRS exploration

**DOI:** 10.3389/fpsyt.2024.1263351

**Published:** 2024-03-04

**Authors:** Emma Gabrielle Dupuy, Thomas Vincent, Catia Lecchino, Annabelle Boisvert, Laurence Trépanier, Sylvie Nadeau, Elaine de Guise, Louis Bherer

**Affiliations:** ^1^ Centre EPIC et centre de Recherche, Montreal Heart Insitute, Montreal, QC, Canada; ^2^ Département de Médecine, Université de Montréal, Montreal, QC, Canada; ^3^ Département de Psychologie, Université de Montréal, Montreal, QC, Canada; ^4^ CRIR—IURDPM, CIUSSS du Centre-Sud-de-l’Île-de-Montréal, Montreal, QC, Canada; ^5^ École de Réadaptation, Université de Montréal, Montreal, QC, Canada; ^6^ Centre de Recherche, Institut Universitaire de Gériatrie de Montréal, Montreal, QC, Canada

**Keywords:** fNIRS, visual art, stress, aging, neurocognition, museum

## Abstract

Recent research suggests that museum visits can benefit psychological well-being by reducing symptoms of stress and anxiety. However, these reported relaxing effects remain inconsistent between studies. Shedding light on the underlying cerebral mechanisms of museum visits might support a better understanding of how it affects psychological well-being. This study aimed to investigate the prefrontal engagement evoked by artwork analysis during a museum visit and to determine if these prefrontal substrates are associated with the museum’s effect on psychological well-being in older adults. Nineteen adults aged between 65 and 79, toured a Baroque-style exhibit at the Montreal Museum of Fine Arts for approximately 20 minutes while equipped with a near-infrared spectroscopy system measuring the prefrontal cortex’s hemodynamic activity. For each painting, participants received the instruction to either (1): analyze the painting and produce a personal interpretation of its signification (*analytic condition*) or (2) visualize the painting without any specific thoughts (*visualization condition*). Questionnaires measuring stress, anxiety, and well-being were administered before and after the visit. Sixteen older women (71.5 ± 4 years) were included in the analyses. Results showed that, at the group level, the *analytic condition* was associated with an increased activation pattern in the left ventrolateral prefrontal region, typically related to attentional processes (not observed in the *visualization condition)*. The activation associated with the *analytic condition* predicted pre-/post-visit reductions in self-reported anxiety and stress in the sample of older women. These observations suggest that the level of engagement of attentional processes during artwork analysis may play a major role in the effect of a museum’s visit on self-reported symptoms of anxiety.

## 1 Introduction

In 2015, the Aging and Health Program of the World Health Organization (WHO) suggested encouraging artistic and cultural practices to foster health in older adults (1). This recommendation is supported by a rising number of scientific publications shedding light on the role of the arts in improving health and well-being. Fancourt and Finn gathered them in an exhaustive scoping review for the WHO in 2019 (2). Among these activities, receptive arts engagement is distinguished from active arts engagement in that it only requires observing, listening, and viewing art pieces, such as theatre, music, and visual art. In older adults, translational and longitudinal research suggests that receptive arts engagement is associated with better mental health and higher indications of well-being (3, 4). Consensual, accessible, and already part of daily life habits for many people, the museum visit gives interesting perspectives on health interventions. Included in a three-month intervention of weekly creative workshops, the museum visit may have contributed to the benefits of the intervention on the mental health and well-being of older adults (5, 6).

Previous research suggests that museum visits can acutely affect visitors’ moods, as well as the subjective experience of stress and its biomarkers (7–9). The calm and restorative environment of the museum, as well as the quality of activity and art collections, may shape psychological well-being outcomes such as concentration and relaxation (10). Hence, the engagement with visual artwork, even passive (i.e., viewing art), may be directly involved in the effects of the museum visit on mood and stress. The preliminary results of a scoping review published by Law et al. (11) showed that viewing artwork might consistently reduce self-reported stress and changes some physiological stress markers, such as systolic blood pressure. This stress reduction would be moderated by important factors, such as the setting in which the artwork is exposed, the artwork itself, the individual characteristics of viewers (e.g., age, gender, art expertise, visit expectations), or the received instructions. A comprehensive understanding of how and by which psychophysiological mechanisms museum activities operate beneficial effects on well-being is paramount to support its use as an effective lifestyle prescription in preventive medicine.

The recent information-processing VIENNA model (12) suggests a continuum of psychological states the viewer encounters when facing visual artwork. These states range from incomprehension and anxiety to feelings of fullness, harmony, and flow. According to this model, a person’s response to visual artwork is influenced by the interaction between the bottom-up processing of artwork features and the top-down influence of viewer intention, memory, or knowledge. Viewing visual artwork would first involve a sequence of bottom-up perceptual processes, engaging the cortical networks related to visual perception (occipital cortex and visual dorsal stream, association area). This first sequence would be associated with a primary affective and emotional response reappraised through a secondary top-down executive process involving the fronto-limbic circuit. During this second sequence, efferent projections of the prefrontal cortex (PFC) to the limbic and parietal regions would support the creation of a coherent meaning with the visual elements and influence the emotion felt by the viewer (12). Investigating prefrontal activity associated with artwork viewing can thus bring some interesting highlights, likely to provide new knowledge about the neurocognitive processes supporting the effect of the museum visit on an individual’s psychological state. Performing this investigation directly in an ecological and artistic environment such as a museum may affect the top-down neurocognitive processes of artwork (13).

In the present study, functional near-infrared spectroscopy (fNIRS), a noninvasive optical imaging technique, was used to perform a primary exploration of prefrontal engagement in analyzing artwork during a museum visit. Then, an examination was conducted to determine whether this prefrontal engagement is associated with changes in subjective stress, anxiety, and well-being after the visit.

## 2 Methods

### 2.1 Participants

Nineteen adults aged between 65 and 79, including eighteen women and one man, participated in fNIRS acquisitions at the Montreal Museum of Fine Arts (MMFA). Participants were recruited from the community through a pool of participants who consented to be contacted for research purposes and by advising members of the MMFA of the research project. To be enrolled, individuals had to be aged between 55 and 85 years old with a normal or corrected vision and audition, be francophone or anglophone, be able to walk with or without technical assistance (e.g., canes and ankle braces), and obtain a score greater than twenty-six on the Mini-Mental State Evaluation. Individuals were excluded if they had a neurological disease history, postural or balance disorders, a recent history of alcohol or substance abuse, reported pain >2/10 on a visual analog scale or had undergone surgery requiring general anesthesia in the last six months.

This study complied with the International Conference on Harmonization Good Clinical Practice (ICH-GCP) and all applicable regulatory requirements. It received the approval of the Centre for Interdisciplinary Research in Rehabilitation of Greater Montreal (CRIR) research ethics board (CRIR-1486-0302). Participants’ consent was collected before assessments.

### 2.2 Procedure

Each participant was invited to the MMFA for an assessment of approximately 60 minutes, including a 20-minute visit to a permanent exhibit. During the visit, the participant was equipped with a wireless fNIRS device (Brite 23, Artinis Medical Systems, Netherlands – 11 detectors, 7 sources, 21 channels, wavelengths: 760 and 850 nm) to measure the hemodynamic activity evoked by visual art processing in the prefrontal cortex. All participants performed the same tour of six paintings in a museum room alone. In front of each painting, the participants were asked to:

(1) *“Analyze the painting”*: look into its elements (characters, landscapes) and its composition, and try to provide a personal interpretation of its meaning (analytic condition);

or

(2) *“Visualize the painting”*: look at the painting without any thoughts and focus specifically on its center or one of its structural elements, such as a specific color dot, for the duration of the trial (visualization condition).

**Figure 1:**
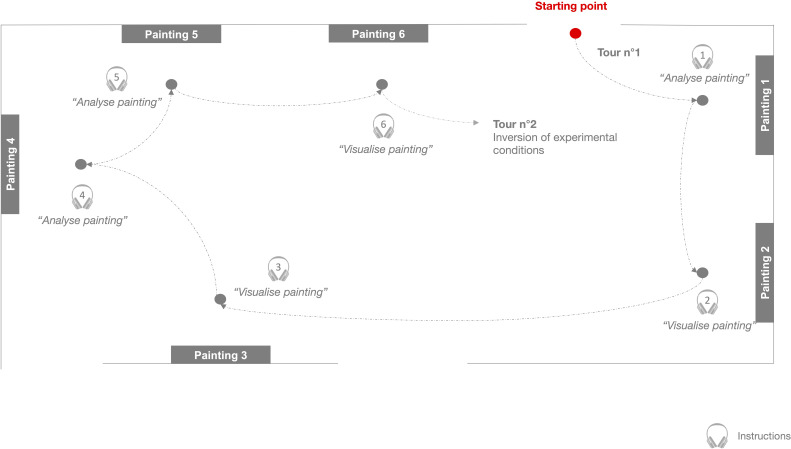
Schematic representation of the timeline for the fNIRS measurement procedure.

They received these instructions through a wireless headset. The details of both instructions were given before the beginning of the tour. Each participant performed the tour of all six paintings twice in the same order with no pause, alternating between visualization and analytic conditions for each painting (e.g., tour 1 – Painting 1: visualization condition, tour 2 – Painting 1: analysis condition). The order of paintings was the same for all participants, but experimental conditions were counterbalanced between participants to minimize carryout effects. In front of the painting, the trial lasted 20 seconds and was followed by a resting period between 20 and 45 seconds (random jittering, average inter-trial interval: 25 s). During each resting period, participants were asked to fix their gaze on the empty spaces of a corner of the room. Participants were then asked to walk to the subsequent painting. Walking duration ranged from 7s to 17s, depending on the physical distance between paintings. The timeline for the fNIRS measurement procedure is schematized in **Figure 1**. Stimulation events were sent using software triggering to synchronize the experimental paradigm with the NIRS signals accurately (14). The paintings were part of the same Baroque collection. This collection was selected for the homogeneity of Baroque production in terms of technique used and visual representation. Also, the Baroque collection was in an easy-to-access museum room near a quiet space for questionnaire completion. All paintings had comparable formats and involved social and non-social representations (i.e., portrait, landscape, mythological pieces). The paintings selected for the tour are presented in the **Supplementary Material.** The visits took place within the regular activities of the MMFA.

Before and after the visit, three auto-administered questionnaires assessed the subjective stress, well-being, and participants’ anxiety state: the Visual Analog Scale of Stress (VAS) (15), the Warwick-Edinburgh Mental Well-Being Scale (WEMWBS) (16), and the State Subscale of State-Trait Anxiety Inventory (STAI-Y) (17).

### 2.3 fNIRS data processing

**Figure 2:**
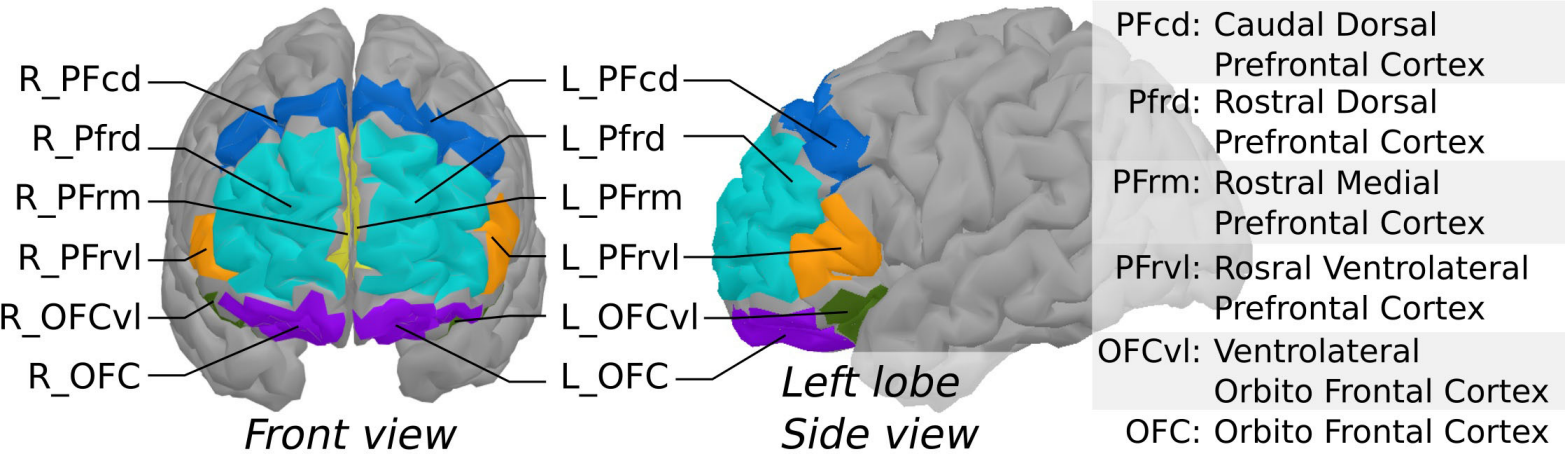
Segmentation of the prefrontal cortex based on MarsAtlas, used to produce region-averages of NIRS task-related effects.

fNIRS data were processed using brainstorm (18) and the nirstorm plugin (github.com/Nirstorm/nirstorm#nirstorm) under Matlab 2017. Signals were first reviewed for major artifacts, and some channels were rejected where heartbeats could not be seen. Participants with too many artefactual channels were discarded (over 50% of the channels). Pre-processing steps were performed in the channel space and comprised motion correction (19) and high-pass filtering with a cut-off of.01 Hz to remove slow varying fluctuations. Channel time series were then projected on the cortical surface of the Colin27 template (20) using the Minimum Norm Estimate algorithm (21). Within-subject t-stat mappings of concentration changes in oxygenated [HbO] and deoxygenated hemoglobin [HbR] evoked by the two experimental tasks (analytic and visualization) were obtained by a first-level Generalized Linear Model (GLM) with a pre-colored noise model (22) applied to the cortical time-series of each subject. The measured variations in [HbO] and [HbR] reflect neurovascular coupling associated with neuronal activity. Regional averages were computed using a coarse version of the MarsAtlas cortical parcellation (23) that consisted of 14 regions, as depicted in **Figure 2**. Lastly, task-specific functional masks were computed from a group-level analysis to keep only the areas potentially engaged in the experimental paradigm. To do so, a second-level GLM with a mixed-effect noise model (22) was applied to produce binary maps from t-stats thresholded at p <.05 (uncorrected). For each experimental condition, this allowed filtering out the regions that elicited no activity at the group level. At the end of this NIRS processing pipeline, within-subject and region-specific effect sizes were used as task-related hemodynamic responses to investigate their relationship with the other study variables in the following statistical analyses.

### 2.4 Data analyses

Wilcoxon signed-rank tests were performed to compare the scores pre-/post-visit from the auto-administered questionnaires (i.e., VAS, WEMWBS, STAI-Y). A delta was computed for each questionnaire by subtracting the pre-visit from the post-visit score (ΔVAS, ΔWEMWBS, ΔSTAI-Y). Using the group-level activation mask (left and right rostral ventrolateral prefrontal cortex, PFrvl), the analytic and visualization conditions’ within-subject effects were integrated into a series of linear hierarchical stepwise regressions against ΔVAS, ΔWEMWBS, ΔSTAI-Y. The regression models were adjusted for the participant’s age (bloc 1) and included the stepwise selection of the HbO or HbR responses in bloc 2. The centrality and normality of the residuals were verified. Neither heteroscedasticity nor multicollinearity was observed. Analyses were performed using SPSS statistics version 28 (IBM Corp, Armonk, New York, USA), and the significance threshold for each test was set at 0.05.

## 3 Results

Two participants of the initial sample of 19 participants were excluded from the analyses because of bad fNIRS signal quality. Another participant was excluded because of the extreme variation in their pre-/post-visit questionnaires, especially in the STAI-Y scores. An increase of 30 pts (from 21 pre-visit to 51 pts post-visit) on his STAI-Y score was observed, against a mean change of -0.56 ± 5.3 pts for the group of participants. Thus, the sample used for the subsequent analyses included sixteen participants, only female, aged 71.5 ± 4 years.

### 3.1 Pre/post-visit changes in questionnaire scores

**Figure 3:**
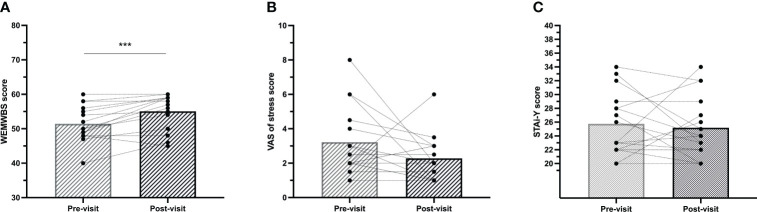
Comparison of scores reported by the participants pre- and post-visit for **(A)** the Warwick-Edinburgh Mental Well-Being Scale (WEMWBS), **(B)** the Visual Analogue Scale (VAS) of stress, and **(C)** the State Subscale of State-Trait Anxiety Inventory (STAI-Y). The height of the bar represents the mean. ***p-value <.005.

Participants showed a significant increase in their WEMWBS score post-visit (55.06 ± 5.1 pts) compared to pre-visit (51.44 ± 5.1) (p = 0.004), indicating a statistically meaningful change in reported well-being (24). No significant changes were observed in the STAI-Y and VAS scores. Pre-/post-visit changes in the STAI-Y, WEMWBS, and VAS of stress scores are displayed in **Figure 3**.

### 3.2 Group-level fNIRS main task effects

**Figure 4:**
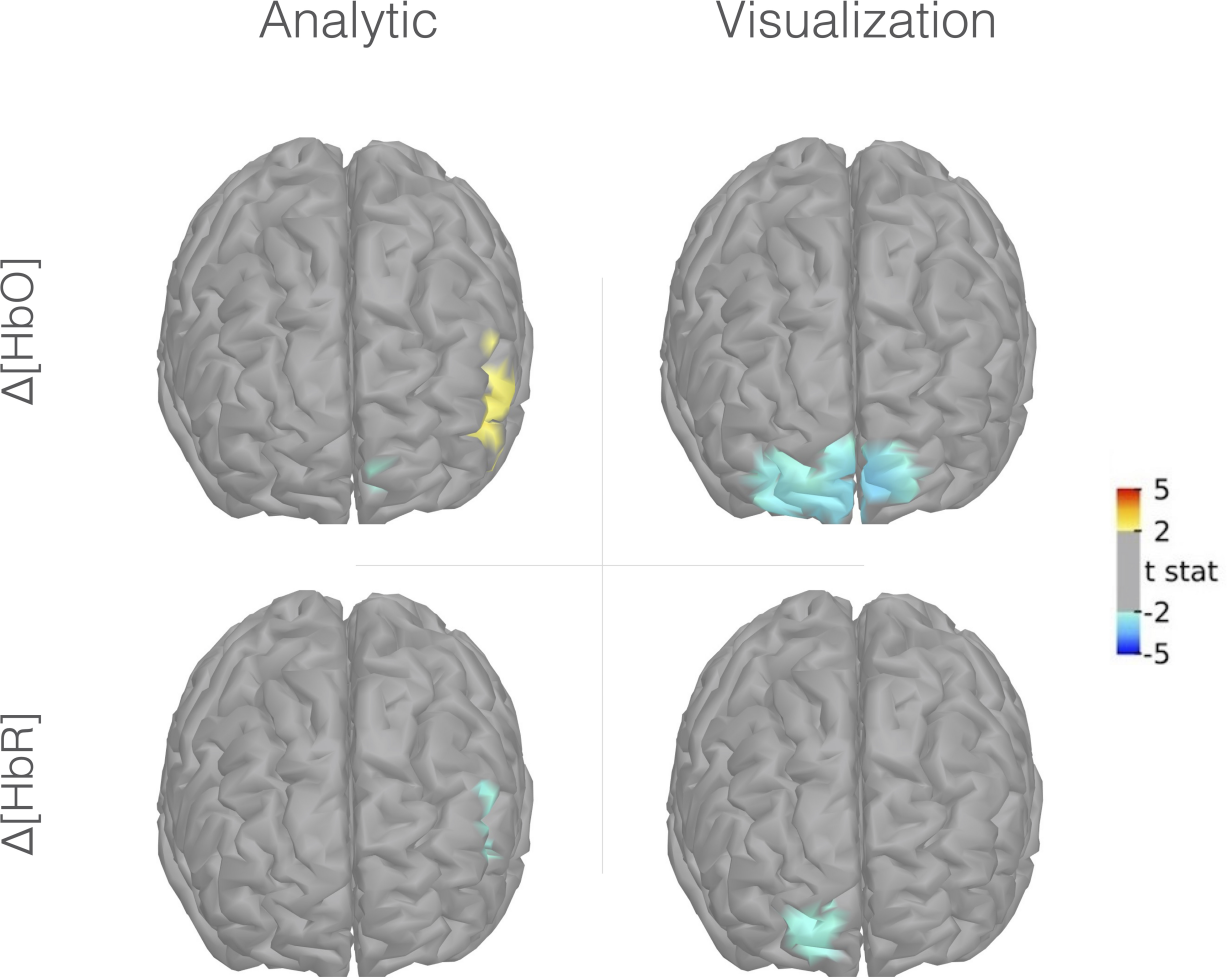
Group-level fNIRS cortical mapping (Colin27 template) of Δ[HbO] (upper portion) and Δ[HbR] (lower portion) in the analytic (right portion) and visualization conditions (left portion). Frontal view, left is right, uncorrected t-stat with a threshold *p-value < 0.05*.

**Figure 4** presents maps of the prefrontal activity evoked by analytic and visualization conditions using the Colin27 template. At the group level, the analytic condition was associated with a prefrontal activity pattern involving a significant increase in HbO and a decrease in HbR concentrations in the left PFrvl, corresponding to typical hyperemia generated by neurovascular coupling. A localized decrease in HbO concentration was also observed in a small cluster of the left rostral dorsal prefrontal cortex (PFrd). A different pattern was observed in the visualization condition. The visualization condition was associated with a bilateral decrease in HbO concentration in the PFrd and a decrease in HbR concentration in the right PFrd. This pattern corresponds to a deactivation process through vasoconstriction. The contrast between analytic and visualization conditions did not reach significance.

### 3.3 Regression-based prediction of pre/post visit changes in questionnaire scores by individual fNIRS task effects

Stepwise regression analyses showed that 40.0% of the variance in the ΔSTAI-Y was predicted by a model including the HbO responses (R2 = 0.40, R2Adj = 0.30, F (1,13) = 4.87, p = 0.046), and 33.0% of the variance in the ΔVAS of stress was predicted by a model including the HbR responses (R2 = 0.33, R2Adj = 0.22, F (1,13) = 4.98, p = 0.044). The activity in the left PFrvl region evoked by the analytic condition was the only significant predictor retained in each model. More precisely, a higher HbO in the left PFrvl during the analytic task was associated with a lower ΔSTAI-Y (β = -3.30; p = 0.046), i.e., a greater decrease in the STAI-Y score after the visit. Also, a higher HbR in the left PFrvl during the analytic task was associated with lower ΔVAS of stress (β = -0.81; p = 0.044), i.e., a greater decrease of the VAS of stress score after the visit. These two models are presented in **Tables 1**, **2**. Regression models performed to predict the variance in the ΔWEMWBS failed to reach statistical significance, but a trend was observed for a model including the HbR responses (R2 = 0.23, R2Adj = 0.12, F (1,13) = 3.98, p = 0.068). This model only included the HbR evoked in the left PFrvl region during the analytic condition, with a higher HbR in the left PFrvl during the analytic task associated with higher ΔWEMWBS (β = 1.16; p = 0.068), i.e., a greater increase of the WEMWBS score after the visit (**Supplementary Table 1**).

**Table 1:** Hierarchical regression predicting the Δ_STAI-Y_ pre-/post visit with age (Step 1), ΔHbO responses selected by a stepwise procedure (Step 2).

Model		β	SD	CI_95_	R^2^; R^2^_Adj_	Model F(df), p
1	Constant	38.91	23.33		0.17; 0.11	F (1,14) = 2.87, p = .112
	Age	-.552	.326	[-1.251,.147]		
	Constant	44.39	20.86		0.40; 0.30	F (1,13) = 4.87, p = .046
2	Age	-.609	.290	[-1.233,.017]		
	**HbO left PFrvl (analysis)**	**-3.288**	**1.49**	**[-6.507, -0.069]**		

β, unstandardized beta coefficient; CI_95_, 95% confidence interval; F(df), degrees of freedom for the F-test; HbO, Oxyhemoglobin; p, p-value; R^2^, R-squared; R^2^_adj_, adjusted R-squared; SD, Standard Deviation.

The bold value indicated a significant association in the regression model (p <0.05).

**Table 2:** Hierarchical regression predicting the Δ_VAS_ pre-/post visit with age (Step 1), ΔHbR responses selected by a stepwise procedure (Step 2).

Model		β	SD	CI_95_	R^2^; R^2^_Adj_	Model F(df), p
1	Constant	9.94	10.86		0.07; 0.00	F (1,14) = 1.00, p = .333
	Age	-.152	.152	[-.478,.173]		
	Constant	4.38	9.90		0.33; 0.22	F (1,13) = 4.98, p = .044
2	Age	-.076	.138	[-.375,.222]		
	**HbR left PFrvl (analysis)**	**-.813**	**.349**	**[-1.601, -0.026]**		

β, unstandardized beta coefficient; CI_95_, 95% confidence interval; F(df), degrees of freedom for the F-test; HbR, deoxyhemoglobin; p, p-value; R^2^, R-squared; R^2^_adj_, adjusted R-squared; SD, Standard Deviation.

The bold value indicated a significant association in the regression model (p <0.05).

## 4 Discussion

This study aimed to explore the prefrontal substrates engaged by the top-down processing of artwork during museum visits and their association with the change in reported well-being after the visit. To do so, the participants were required to analyze the selected paintings by breaking down their visual content (character, landscape) and producing a personal interpretation of their significance while being equipped with an fNIRS. The results demonstrated that analyzing painting engages a consistent pattern of prefrontal activity across participants. This pattern engages the left PFrvl, a region typically associated with attentional and cognitive control processes. Such a pattern of prefrontal activity was not observed when participants were required to visualize the painting (control task), which evoked a bilateral deactivation of the PFrd. According to the regression analyses, the activity of the left PFrvl associated with the analysis of paintings might support the reduction of self-reported stress and anxiety symptoms in older adults after the visit, as assessed by the pre/post visit variations in STAI-Y and VAS scales. The regression models predicted 40% and 33% of the variance in the pre-/post-visit changes of self-reported anxiety and stress, respectively, with increased left PFrvl activity in the analytic condition associated with a post-visit reduction of these symptoms. These models excluded the activity engaged by the mere painting visualization. Together, these observations suggest that the top-down cognitive control processes might play a critical role regarding the extent to which museum visits affect older adults’ psychological well-being.

To our knowledge, the top-down neurocognitive processing of artwork has scarcely been done before in a museum context. Yet, exposure to original paintings in an ecological and artistic environment, such as a museum, is likely to affect the appraisal of paintings’ aesthetic qualities and influence the viewers’ expectations. Most of the previous research performed in the field, including those using fNIRS (25, 26), was done in-lab, required participants to be seated or lengthened, and pieces of art resumed a screen projection. In daily life, the contact with artwork is likely to take place in a privileged and/or collective spaces such as a museum, interacting with an individual in movement, sometimes in interaction himself, and presenting a piece of art in relief, embedded in a context that might magnify its aesthetic dimension. These contextual factors are likely to affect the psychological effect of art exposure and top-down processes involved in art viewing. Previous research demonstrated that contextual information introducing a picture as a piece of art favors a so-called “aesthetic” processing mode (13, 27, 28). This processing mode would enable a subjective and perceptive experience of the artwork, engaging the viewers’ attention while other objects, events, and everyday concerns would be suppressed (29). A recent model [i.e., the VIENNA model, (12)] called this mode of processing a “flow state,” which would correspond to an effortless concentration associated with a feeling of aesthetic, emotional harmony. Functional magnetic resonance neuroimaging studies showed that viewing oriented on aesthetic (i.e., concentrating on the mood, color, shape of the painting, and the feeling evoked) engages the left lateral prefrontal areas (28). This observation merges with the pattern of activity observed in the present study, suggesting a potential overlap between cognitive control processes tied to painting analysis and the aesthetic viewing orientation fostered by Cupchick et al. (28),. According to these authors (28), this activation would be tied to the self-referential nature of aesthetic perception, which requires maintaining attention on internally generated cognitions (i.e., endogenous attention) (30, 31). Such endogenous attention might have also been involved in analyzing the painting performed here, during which the participant had to concentrate on pictural elements to provide a personal interpretation. Hence, the engagement of PFrvl in painting analysis may be linked to the involvement of mnemonic processes aiming to retrieve and select the stored knowledge relevant to interpreting the painting’s elements and symbolism (31).

Furthermore, the left PFrvl engagement level in the analytic condition is associated with the reduction of stress and anxiety following the museum visits. This suggests a linear relationship between the cognitive processes engaged by the viewer in painting analysis and the extent to which the museum visit affects perceived stress and anxiety. Previous research assessing fNIRS activity evoked by artwork viewing reported a consistent pattern of prefrontal activity when participants were asked to interpret the artist’s emotions (25). Much evidence collected during the past decades identifies the lateral prefrontal cortex as a key brain structure involved in fronto-limbic pathways linked to bidirectional interaction between emotion and cognition (32–34). Kirk et al. (35) observed its engagement in art-related emotional regulation processes. More precisely, they reported a strong coupling between PFrvl and the amygdala when an individual faces an image with a high emotional valence (e.g., fearful image), presented as an artwork instead of a real-life event picture. This result was interpreted as the engagement of a top-down appraisal process that would inhibit innate emotional response (i.e., utilitarian emotions, fight-flight) to allow a more distanced and reflective perspective. Consistently, our results suggest that when participants are involved in a task requiring a reflective perspective on the painting, the level of engagement of attentional processes might be involved in emotional response modulation, a mechanism likely to moderate the stress-reducing effect of museum visits.

If the small sample size may have contributed to the absence of a pre-/post-visit decrease in self-reported stress and anxiety, this lack of significant changes supports the existence of potential moderating factors in the stress-reducing effect of the museum visit (10). It has been recently proposed that the stress-reducing benefits of visual art stimulation may be due to its capacity to distract the viewer from their stress (10). However, our results suggest that more than a simple distraction, an active engagement towards the artwork might play a moderating role in reducing perceived stress and anxiety symptoms observed after the visit. This observation challenges the notion of museum visits as a receptive art engagement when considering its potential to affect the psychological state acutely. Recent research in art-health museum practices, such as art therapy and museum education, attributes their benefits for psychological well-being to the capacity of artwork to create meaningful connections with individuals’ emotions, past, and memories (36). Our observations support this assumption but suggest that this capacity may fluctuate between individuals. Hence, further explorations are needed to confirm these preliminary observations and shed light on the factor moderating an active engagement of the viewer with the artwork and if we can facilitate it with museum mediation or additional information. Such a conclusion might support future development in art-health museum practices, such as the “museum prescription” approach, by fostering the neurocognitive processes involved in their effect on well-being.

Some important elements must be considered to bring an accurate conclusion to this study, reflecting the scope of these results and their perspectives. First, the recruiting procedure of this study did not achieve parity between women and men. Exclusively composed of women, the final sample of participants restrains the generalization of the above-mentioned observations. Indeed, aesthetic preferences might differ between women and men. Previous research showed that, compared to men, women tended to find more pleasing and relaxing representational art with soft edges and smooth contours, such as impressionist and baroque paintings (37–39). Hypothetically, this observation would be due to sex differences in visuospatial abilities, with women tending to preferentially process categorical spatial relations while men process coordinate spatial relations (40). However, as these studies compare individuals based on their biological sex, we cannot exclude that psychosocial aspects fluctuating with gender roles (e.g., education, cultural references, tolerance for uncertainty) may have contributed to these observations. This last assumption is supported by the fact that sex-related differences in terms of aesthetic preference were not observed in women and men with a particular familiarity or expertise in visual art (38, 40). In the present study, the recruitment strategy involved reaching MMFA and community members who are likely to have heterogeneous degrees of familiarity with visual art. Yet, prior knowledge about the art object shapes its visual exploration, orients attention, and seems to favor aesthetic appraisal (41). Controlling or measuring the degree of participants’ expertise and familiarity with visual art might have provided relevant information for interpreting fNIRS results. However, this study selected only baroque representational paintings that are rather accessible, limiting the potential bias related to the interaction between participants’ expertise and abstract art appreciation (42). A replication of this experimental paradigm with other painting styles or assessing observer expectations would be particularly interesting to further investigate the influence of top-down and bottom-up processes on psychological well-being. Finally, future studies may consider including the measurement of physiological stress markers (e.g., salivary cortisol samples or heart rate) to understand the psychophysiological mechanisms underlying the stress-reducing benefits of the museum visit.

Performed directly in the museum environment, this neuroimaging study gives new insight into the neurocognitive processes that support the effect of museum visits on psychological well-being. They suggest that the level of engagement of attentional processes during artwork analysis may play a major role in the effect of a museum’s visit on self-reported symptoms of anxiety and stress, at least in older women. These preliminary observations pave the way for future investigations aiming to identify the determinants of active engagement against the artwork during the museum visits and their potential to increase the visit’s effect on psychological well-being.

## Data availability statement

The raw data supporting the conclusions of this article will be made available by the authors, without undue reservation.

## Ethics statement

The studies involving humans were approved by Centre for Interdisciplinary Research in Rehabilitation of Greater Montreal (CRIR) research ethics board (CRIR-1486-0302). The studies were conducted in accordance with the local legislation and institutional requirements. The participants provided their written informed consent to participate in this study.

## Author contributions

EGD: Conceptualization, Formal analysis, Investigation, Supervision, Writing – original draft, Project administration, Funding acquisition. TV: Conceptualization, Data curation, Formal analysis, Investigation, Methodology, Software, Supervision, Visualization, Writing – review & editing. CL: Conceptualization, Investigation, Writing – review & editing. AB: Investigation, Writing – review & editing. LT: Investigation, Writing – review & editing. SN: Conceptualization, Funding acquisition, Investigation, Project administration, Resources, Supervision, Validation, Writing – review & editing. EdG: Conceptualization, Data curation, Funding acquisition, Investigation, Methodology, Project administration, Resources, Supervision, Validation, Writing – review & editing. LB: Conceptualization, Funding acquisition, Methodology, Project administration, Resources, Supervision, Validation, Writing – review & editing.
