# Supplementary material for: Prefrontal engagement predicts the effect of museum visit on psychological well-being: an fNIRS exploration
Source: Front Psychiatry. 2024 Mar 4;15:1263351. doi: 10.3389/fpsyt.2024.1263351 (PMC10944881; doi:10.3389/fpsyt.2024.1263351)
Supplement: Supplementary file 1 [file DataSheet_1.pdf]

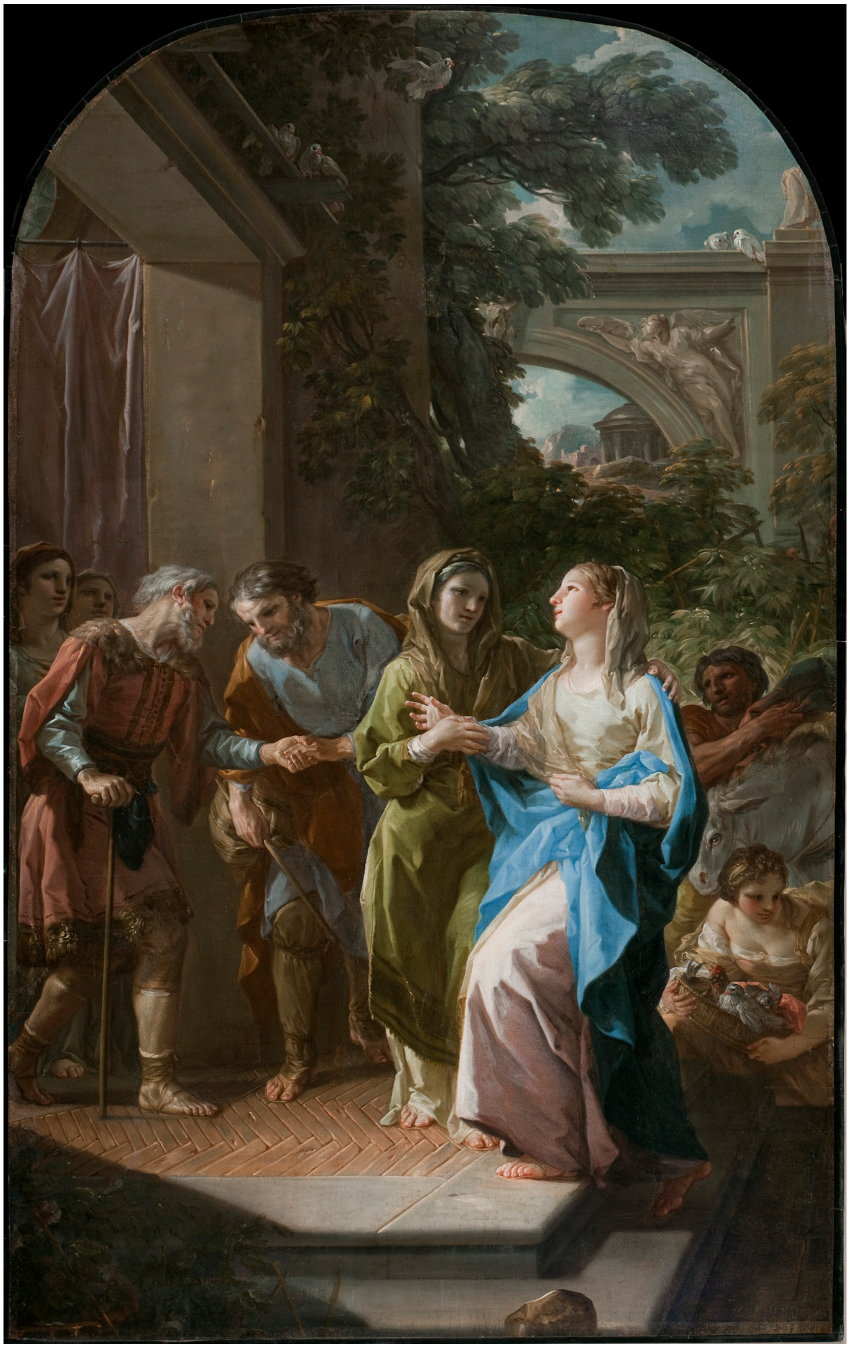

Corrado Giaquinto
Molfetta, Italy, 1703 – Naples 1766
The Visitation
1764 1765
Oil on canvas
287 x 177.8 cm
The Montreal Museum of Fine Arts, purchase,
Horsley and Annie Townsend Bequest
Photo MMFA, Christine Guest

François de Troy
Toulouse 1645 – Paris 1730
Presumed Portrait of Madame
de Franqueville and Her Children
1712
Oil on canvas
138.5 x 163.4 cm
The Montreal Museum of Fine Arts,
gift of Mr. and Mrs. Michal Hornstein
Photo MMFA

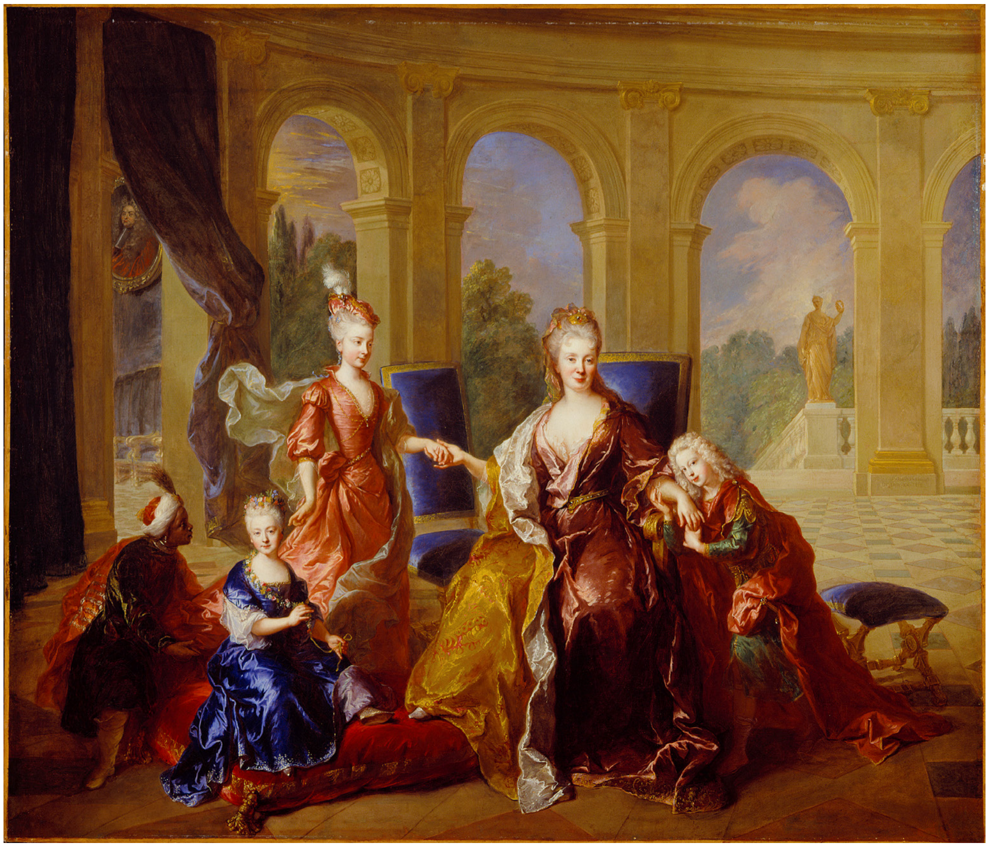

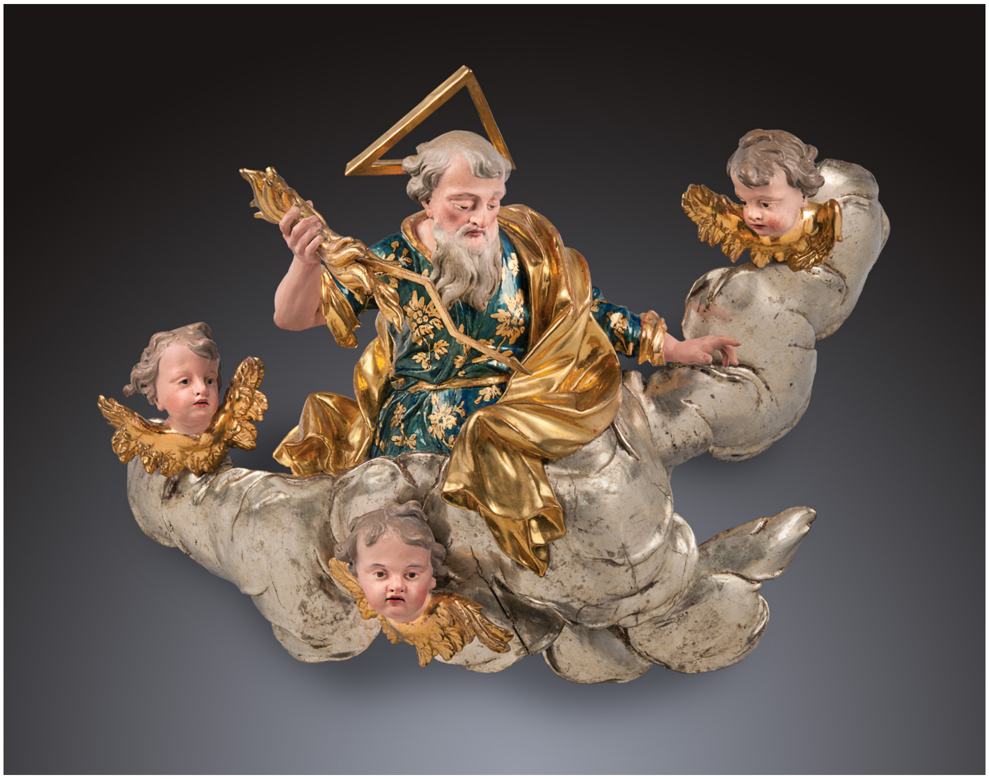

Austria School, Vienna
God the Father and Three Angel Heads
About 1750 1775
Polychromed wood
84.4 x 104.1 x 58.4 cm
The Montreal Museum of Fine Arts,
purchase, Horsley and Annie Townsend
Bequest
Photo MMFA, Christine Guest

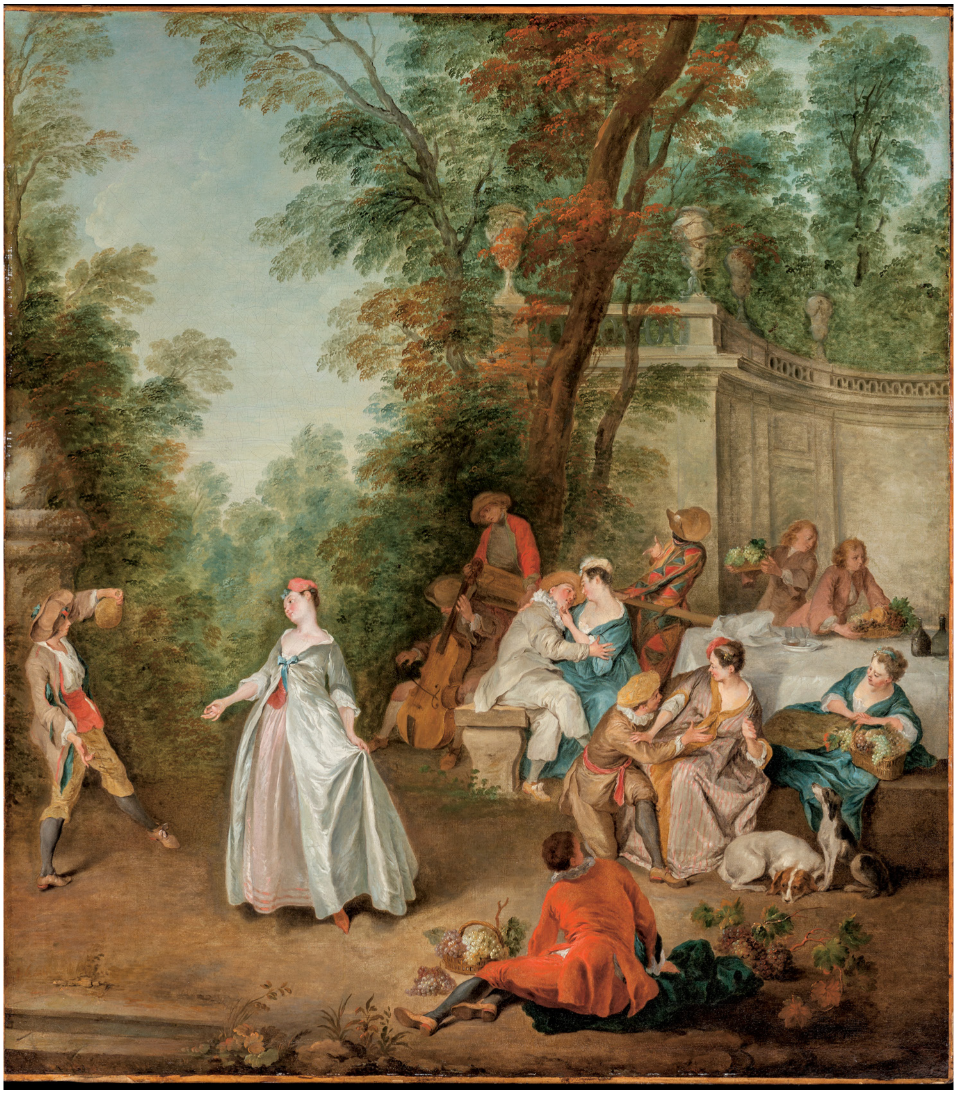

Nicolas Lancret
Paris 1690 – Paris 1743
Autumn
About 1725 1730
Oil on canvas
100 x 89 cm
The Montreal Museum of Fine Arts, purchase, F. Cleveland Morgan Fund and the Museum Campaign 1988 1993 Fund
Photo MMFA, Christine Guest

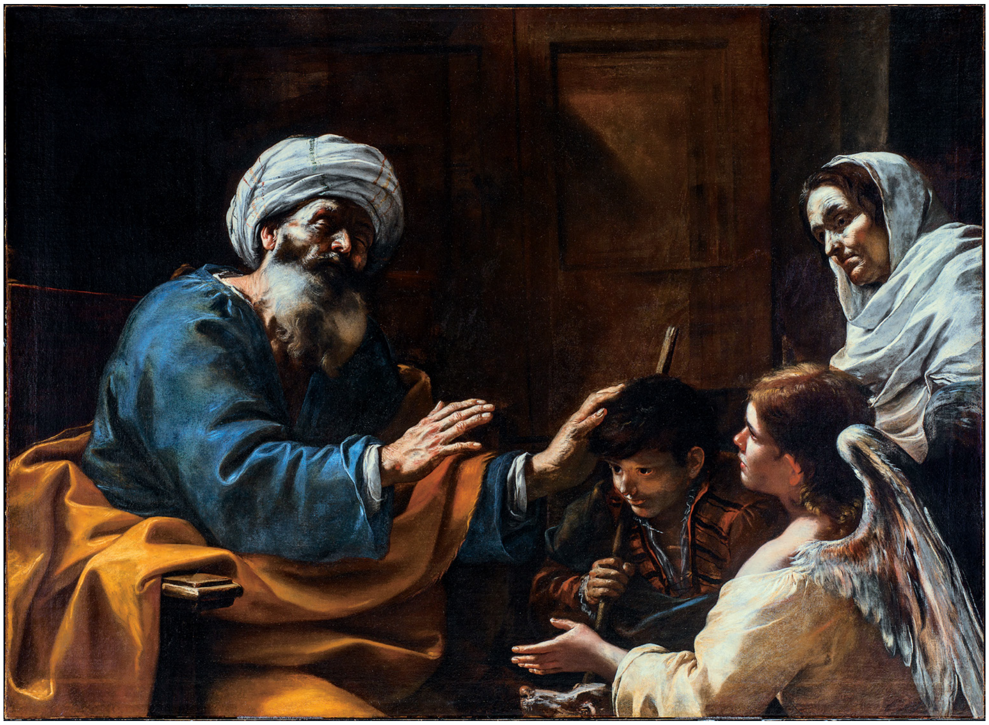

Mattia Preti
Taverna, Italy, 1613 – Valletta, Malta, 1699
Tobit Blessing Tobias
About 1660
Oil on canvas
130.3 x 180.2 cm
The Montreal Museum of Fine Arts, purchase,
Horsley and Annie Townsend Bequest
Photo MMFA, Christine Guest

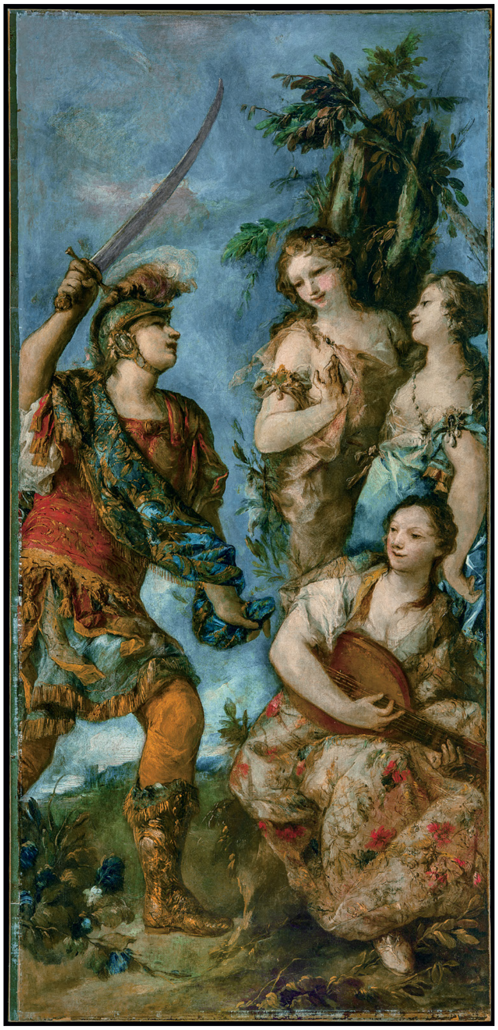

Gianantonio Guardi
Vienna 1699 – Venice 1760
Francesco Guardi
Venice 1712 – Venice 1793
Rinaldo and the Nymphs
About 1745 1755
Oil on canvas
250 x 121 cm
The Montreal Museum of Fine Arts, purchase
in tribute to Michal and Renata Hornstein
Photo MMFA, Christine Guest
