# Supplementary material for: Prefrontal engagement predicts the effect of museum visit on psychological well-being: an fNIRS exploration
Source: Front Psychiatry. 2024 Mar 4;15:1263351. doi: 10.3389/fpsyt.2024.1263351 (PMC10944881; doi:10.3389/fpsyt.2024.1263351)
Supplement: Supplementary file 2 [file DataSheet_2.pdf]

Supplementary table 1. Hierarchical regression predicting the ΔWEMWBS pre-/post visit with age (Step 1), ΔHbR responses selected by a stepwise procedure (Step 2).

Model		β	SD	CI_{95}	R^2; R^2_{Adj}	Model F(df), p
1	Constant	3.03	16.84		0.00; -0.07	F(1,14) = .001, p = .972
	Age	.008	.235	[-.496, .513]		
2	Constant	10.93	15.80		0.23; 0.12	F(1,13) = 3.98, p = .068
	Age	-.100	.220	[-.576, .376]		
	HbR left PFvl (analysis)	1.158	.581	[-.097, 2.414]		

Notes. β: unstandardized beta coefficient, CI_{95}: 95% confidence interval, F(df): degrees of freedom for the F-test, HbR: deoxyhemoglobin, p: p-value, R^2:R-squared, R^2_{adj}: adjusted R-squared, SD: Standard Deviation.
